# Supplementary material for: Probing hyperbolic and surface phonon-polaritons in 2D materials using Raman spectroscopy
Source: Nat Commun. 2023 Jul 11;14:4098. doi: 10.1038/s41467-023-39809-3 (PMC10336144; doi:10.1038/s41467-023-39809-3)
Supplement: Supplementary file 1 — Supplementary Information [file 41467_2023_39809_MOESM1_ESM.pdf]

Supplementary Information: Probing Hyperbolic and Surface Phonon-Polaritons in 2D Materials Using Raman Spectroscopy

Alaric Bergeron,[1] Clément Gradziel,[1] Richard Leonelli,[2] and Sébastien Francoeur[1,*]

[1]*Département de génie physique, Polytechnique Montréal, Montréal, Québec H3C 3A7, Canada*

[2]*Département de physique & Regroupement Québécois sur les Matériaux de Pointe (RQMP), Université de Montréal, Montréal, Québec, H3C 3J7, Canada*

1. Vibrational modes

GaSe has a hexagonal layered lattice structure with each monolayer consisting of two opposed tetrahedral layers of gallium and selenium in a Se-Ga-Ga-Se sequence. The unit cell of the monolayer contains four atoms and the resulting 12 vibrational modes are identified using the representations of the $D_{3\mathrm{h}}$ point-group.

The monolayer stacking order determines the bulk polytype. The most common polytype identified in the literature is ϵ-Gase. It expresses a ABA stacking order and a $D^1_{3\mathrm{h}}$ space group, and it lacks inversion symmetry [1–5]. The γ-GaSe ($C^5_{3\mathrm{v}}$) and β-GaSe polytypes ($D^4_{6\mathrm{h}}$) have also been identified by some authors. Due to the weak inter-layer interactions, all polytypes exhibit similar electronic and vibrational characteristics[4, 6–8] and it can be challenging to distinguish them. In Raman spectroscopy for example, the splittings resulting from the stacking order is typically smaller than the phonon linewidths. Large and high quality Bridgeman-grown crystals such as those used in this work are predominantly of the ϵ polytype [1–5].

The unit cell of ϵ-GaSe spans two monolayers and contains 8 atoms. There are a total of 24 vibrational modes. The bulk normal modes bear the same symmetry representations as those of the monolayer, but each mode is now split into Davydov doublets[2], where the two modes are built from two monolayer modes placed either in- or out-of-phase. Owing to the very weak interlayer interactions in GaSe [2, 9, 10], the Davydov doublets are nearly degenerate and, for the high-energy optical modes studied in this work, have yet to be resolved. Supplementary Table 1 lists the 12 *in-phase* optical normal modes of bulk $\epsilon-$GaSe along with their reported energies and selection rules. The remaining 12 *out-of-phase* modes can be constructed by inverting the displacement in one of the two monolayers.

2. Momentum conservation in Raman scattering

Momentum conservation requires $\boldsymbol{k} = \boldsymbol{q_i} - \boldsymbol{q_s}$ or $k = \sqrt{q_i^2 + q_s^2 - 2q_iq_s\cos(\psi)}$, where $\boldsymbol{q}_i$ and $\boldsymbol{q}_s$ are the incident and scattered photon wavevectors, and ψ is the angle between these two vectors. Hence, Raman spectroscopy can probe excitations with wavevector magnitudes k in the range $|q_i - q_s| \leq k \leq q_i + q_s$. Using visible light to probe material excitations, the magnitude of q_i and q_s are of the order of $10^5\,\mathrm{cm}^{-1}$.

Supplementary Table 1. Normal vibrational modes of ϵ-GaSe. For each normal mode, from top to bottom are the Mulliken symbols, schematic atomic displacements within the unit cell, frequencies, mode activity, and Raman selection rules. The mode frequencies, along with their attribution, are averaged frequencies found in Refs. [1, 11–15]. Raman selection rules are reported using the Porto notation.

$E'(1^2)$	$A_2''(1^2)$	$E''(1^{1,2})$	$A_1'(1^{1,2})$	$E''(2^{1,2})$	$E'(2^{1,2})$	$A_2''(2^{1,2})$	$A_1'(2^{1,2})$
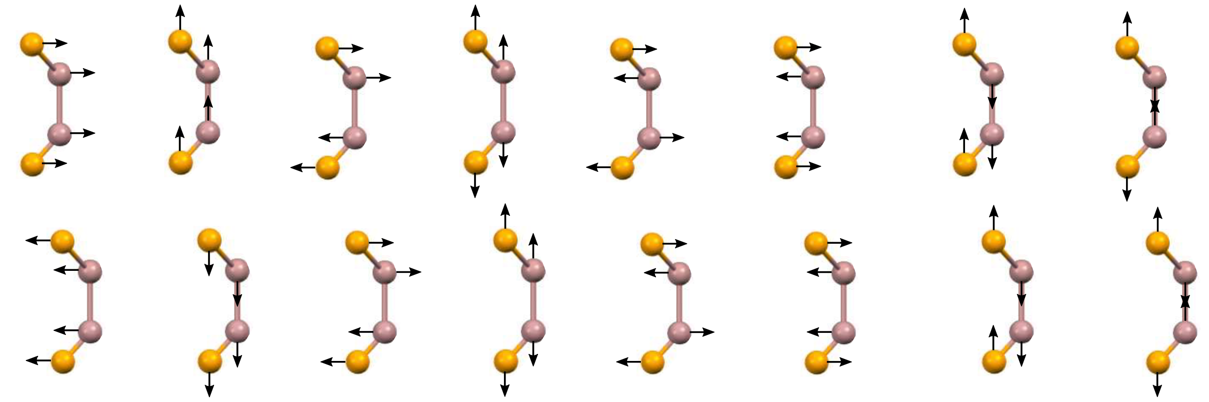							
$19.5\,\mathrm{cm}^{-1}$	$37.0\,\mathrm{cm}^{-1}$	$60.1\,\mathrm{cm}^{-1}$	$134.2\,\mathrm{cm}^{-1}$	$210.4\,\mathrm{cm}^{-1}$	TO:$213.5\,\mathrm{cm}^{-1}$<br>LO:$253.3\,\mathrm{cm}^{-1}$	TO:$236.5\,\mathrm{cm}^{-1}$<br>LO:$246.1\,\mathrm{cm}^{-1}$	$307.6\,\mathrm{cm}^{-1}$
Raman<br>Infrared	Infrared	Raman	Raman	Raman	Raman<br>Infrared	Infrared	Raman
-	-	$\bar{x}(yz)x$<br>$\bar{y}(xz)y$	$\bar{x}(yy)x$<br>$\bar{x}(zz)x$<br>$\bar{y}(xx)y$<br>$\bar{y}(zz)y$<br>$\bar{z}(xx)z$<br>$\bar{z}(yy)z$	$\bar{x}(yz)x$<br>$\bar{y}(xz)y$	$\bar{y}(xx)y$ [LO]<br>$\bar{x}(yy)x$ [TO]<br>$\bar{z}(xx)z$ [TO]<br>$\bar{z}(yy)z$ [TO]	-	$\bar{x}(yy)x$<br>$\bar{x}(zz)x$<br>$\bar{y}(xx)y$<br>$\bar{y}(zz)y$<br>$\bar{z}(xx)z$<br>$\bar{z}(yy)z$

For typical phonon frequencies ($\omega \approx 200{-}1000\,\mathrm{cm}^{-1}$), bulk polaritonic effects are observed for wavevectors values of the order of $k \sim 5\times10^3\,\mathrm{cm}^{-1}$[16]. Such low value can only be achieved using a near-forward measurement configuration, $\psi \approx 0$ (see Fig. 1(b)). Although near-forward Raman scattering can be implemented relatively easily, it is rarely used and the large majority of Raman instruments are configured to be operated in a backward scattering configuration only. In the commonly used backscattering configuration (see Fig. 1(a)), $\psi \approx 180^\circ$ and the smallest wavevector probed is $k \approx 10^5\,\mathrm{cm}^{-1}$. Although this value is much less than the overall size of the Brillouin zone, it is nonetheless quite far from the

light line where the coupling between a polar phonon and light is significant in bulk samples. Hence, only purely mechanical vibrational modes (phonons) are generally observed in a backscattering configuration. It is important to note that, in some conditions, near-forward Raman scattering can be observed in a backward scattering configuration. 1(c) illustrated the case where the laser excitation is reflected at the back surface and redirected towards the collection objective. Forward-scattering can then be observed in the Raman spectra. This aspect is further discussed in Supplementary Notes 11 and 12 for quasi-bulk GaSe sample with thicknesses up to 70 µm.

In the context of polaritons in thin Van der Walls crystals, two significant aspects influence the magnitude of polariton wavenumbers. First, the crystal anisotropy provides an hyperbolic dispersion relation in the reststrahlen region, such that there are no upper limit on the magnitude of k. Second, increasing confinement by reducing sample dimensions allows taking advantage of this hyperbolicity by pushing polariton wavenumbers to higher values. The deep subwavelength confinement in submicron size samples pushes k values in a range compatible with backscattering Raman spectroscopy (Supplementary Figure 1(a)).

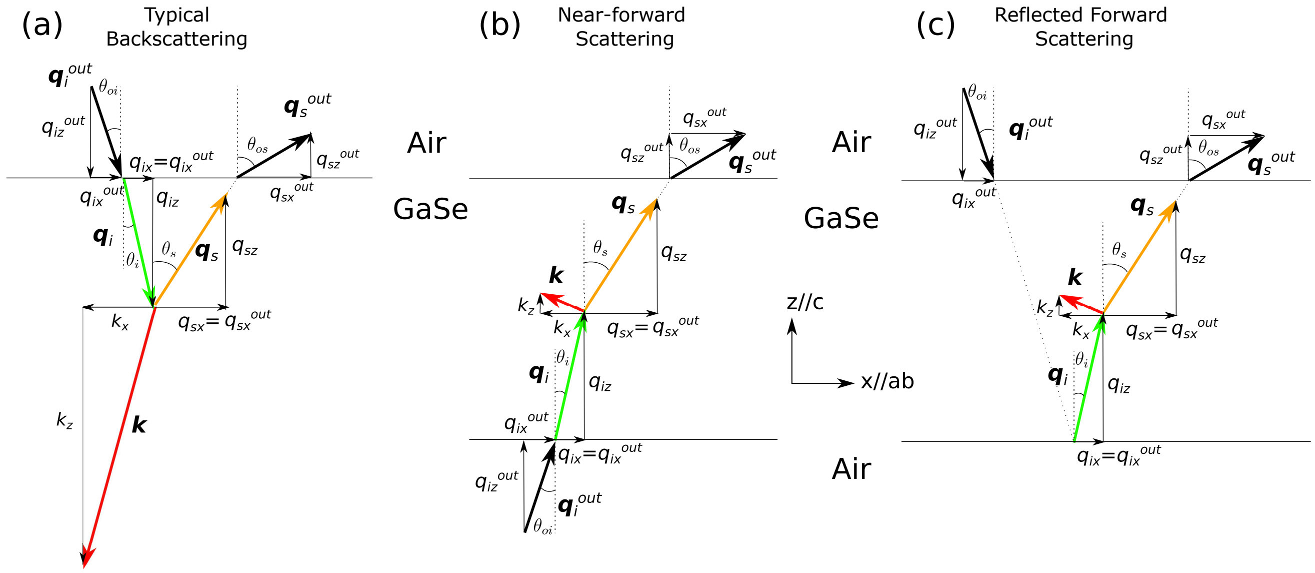

Supplementary Figure 1. Schematic representation of the three relevant Raman scattering geometries. $\boldsymbol{q_i}$, $\boldsymbol{q_s}$ and $\boldsymbol{k}$ represent the incident, scattered, and polariton wave vectors, respectively. θ and θ_o are the angles of the incident and scattered beams relative to the z or c axis. The subscript o is added to propagation angles outside the sample. $\boldsymbol{q_i}^{out}$ and $\boldsymbol{q_s}^{out}$ are the photon wavevectors outside the sample. (a) Typical backscattering configuration, where the scattered photon is redirected toward the upper surface of the sample by the large vertical component k_z of the probed polariton wave vector. (b) Near-forward geometry, where the scattered photon's propagation direction is only slightly altered by the small $\boldsymbol{k}$ and exits the sample through the opposing face. (c) Example of reflected forward scattering, wherein the incident photon is first reflected by the lower surface of the sample, allowing near-forward scattering to be observed in a backscattering experimental configuration.

3. Phonon dispersion in uniaxial crystals

In uniaxial crystals, the energy of polar phonons is determined by the direction of atomic movements with respect to the extraordinary axis (A or E modes) and the long-range coulomb interaction at the origin of the LO and TO splitting. For propagation along high symmetry crystal directions, phonons can straightforwardly be defined by their A, E, TO and LO characters. At oblique angles however, phonons exhibit mixed characters and reduced symmetry[17] and, depending on the relative magnitudes of the electrostatic and crystalline

contributions, dispersion relatively close to the Brillouin zone center can be observed.[18–20] In GaSe, the Coulomb interaction largely dominates the crystal anisotropy and polar modes preserve a dominant TO or LO characters at all angles. As a function of internal propagation angle θ, the photon frequency is given by [1, 19]

$$\omega_{To}^2(\theta) = \omega_{\mathrm{TO}\perp}^2, \tag{1}$$

$$\omega_{Te}^2(\theta) = \omega_{\mathrm{TO}\perp}^2 \cos^2\theta + \omega_{\mathrm{TO}/\!/}^2 \sin^2\theta, \tag{2}$$

$$\omega_{Le}^2(\theta) = \omega_{\mathrm{LO}/\!/}^2 \cos^2\theta + \omega_{\mathrm{LO}\perp}^2 \sin^2\theta. \tag{3}$$

and illustrated in Fig. 2(a). The three phonon dispersion branches can be identified as the transverse ordinary phonon (To, $E'_{\mathrm{TO}} \rightarrow E'_{\mathrm{TO}}$), transverse extraordinary phonon (Te, $E'_{\mathrm{TO}} \rightarrow A''_{2\mathrm{LO}}$) and longitudinal extraordinary phonon (Le, $A''_{2\mathrm{LO}} \rightarrow E'_{LO}$).

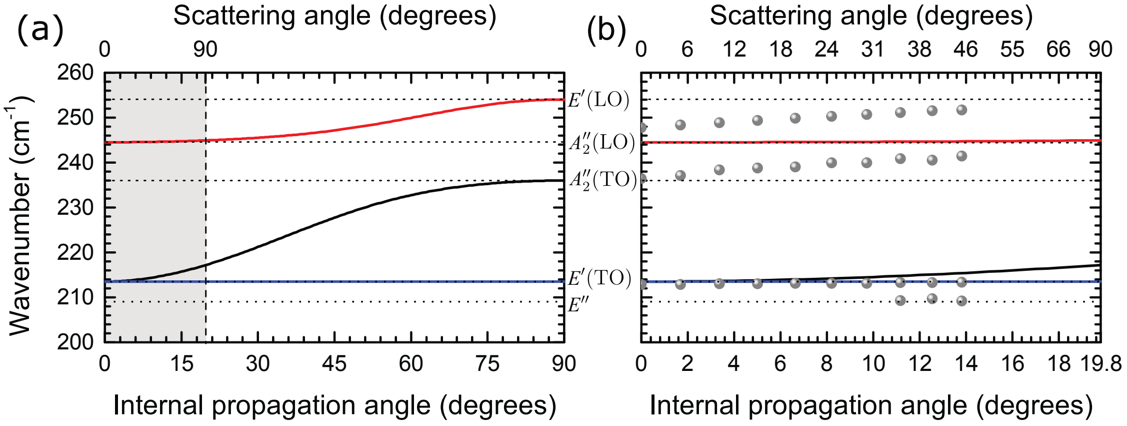

Supplementary Figure 2. Comparison between the calculated lattice-induced dispersion and observed angular dispersion. (a) Complete lattice dispersion curve of zone-center phonons ($\omega/c \ll k \ll \frac{\pi}{a}$) as a function of internal propagation angles with respect to the uniaxe c. Red and black curves illustrate the extraordinary phonon branches, and the ordinary non-dispersive To branch is represented by the blue curve. The upper scale indicates the corresponding external optical scattering angle, with the shaded area illustrating the accessible internal propagation angles below the total internal reflection angle of 19.8°. Lattice mode energies are indicated by the horizontal dotted lines. (b) Enlarged view of the shaded region in (a), with the measured data points shown in Fig. 2(f) of the main text. Uncertainties are smaller than the data points.

As shown in Fig. 2(b), the experimental data from the 650-nm thick sample presented in

the main text is incompatible with the expected position and dispersion of purely mechanical phonons.

4. Permittivity of GaSe

For materials with multiple polar phonons, the permittivity involves the coupling of the n_λ polar modes occurring along the direction λ and can be defined as [21],

$$\epsilon_\lambda(\omega) = \epsilon_{\lambda\infty} \prod_{i=1}^{n_\lambda} \left(\frac{\omega^2_{\mathrm{LO}\lambda i} - \omega^2}{\omega^2_{\mathrm{TO}\lambda i} - \omega^2} \right), \tag{4}$$

where ϵ_∞ represents the high-frequency permittivity, and ω_{TO} and ω_{LO} are the transverse TO and LO phonon frequencies. For GaSe, there are two distinct groups of three polar modes $2 \times (1\mathrm{A} + 2\mathrm{E})$. The first group consists of out-of-phase acoustic modes (see Supplementary Table 1) and occur at very low frequencies. Hence, these weak resonances [8, 22] at low-frequency do not appreciably affect the permittivity in the vicinity of the second group of polar modes, located above $200\,\mathrm{cm}^{-1}$. Hence, for ordinary (transverse) polaritons, the permittivity is given by,

$$\epsilon_\perp(\omega) = \epsilon_{\infty\perp} \left(\frac{\omega^2_{\mathrm{LO}\perp} - \omega^2}{\omega^2_{\mathrm{TO}\perp} - \omega^2} \right), \tag{5}$$

and, for extraordinary polaritons propagating in the ab plane, the permittivity is

$$\epsilon_\parallel(\omega) = \epsilon_{\infty\parallel} \left(\frac{\omega^2_{\mathrm{LO}\parallel} - \omega^2}{\omega^2_{\mathrm{TO}\parallel} - \omega^2} \right). \tag{6}$$

In these equations, the damping factor has been omitted for simplicity. It can be reintroduced through the following substitution $\omega^2 \to \omega^2 + i\Gamma\omega$. The parameters used to model the permittivity were taken from Ref. 23 and are listed in Supplementary Table 2.

Supplementary Table 2. Transverse and longitudinal frequencies and high-frequency permittivities for the in-plane and out-of plane polar phonons in GaSe [23].

Mode (direction)	ω_{TO} (cm^{-1})	ω_{LO} (cm^{-1})	ϵ_∞
In-plane $E'(2^{1,2})$ mode ($\perp$)	213.5	254	7.44
Out-of-plane $A''_2(2)$ mode ($\parallel$)	236	244.5	5.76

The general dispersion relation for arbitrary wave vector directions of extraordinary waves polaritons depends on both the wavelength and the propagation angle θ relative to the c axis [19],

$$\frac{k^2c^2}{\omega^2} = \epsilon_e(\omega,\theta) = \frac{\epsilon_\perp(\omega)\epsilon_\parallel(\omega)}{\epsilon_\perp(\omega)\sin\theta^2 + \epsilon_\parallel(\omega)\cos\theta^2}, \tag{7}$$

which can also be expressed as

$$\frac{\omega^2}{c^2} = \frac{k_\perp^2}{\epsilon_\perp} + \frac{k_\parallel^2}{\epsilon_\parallel}. \tag{8}$$

For positive $\epsilon_\perp$ and $\epsilon_\parallel$, the dispersion relation (8) describes a revolution ellipsoid. For negative $\epsilon_\perp$ and positive $\epsilon_\parallel$, the dispersion relation describes a one-sheeted revolution hyperboloid, known as a Type I hyperbolic dispersion. For negative $\epsilon_\perp$ and positive $\epsilon_\parallel$, the dispersion describes a two-sheeted revolution hyperboloid, known as Type II hyperbolic dispersion (yellow regions in Fig. 1). Frequency regions where both permittivities are negative correspond to a *double* Reststrahlen region (blue regions in Fig.1).

5. Raman tensors

The Raman tensor components for each of the normal modes of are [24]:

$$\begin{aligned}
\overleftrightarrow{R}_{A_1'} &= \begin{pmatrix} a & 0 & 0 \\ 0 & a & 0 \\ 0 & 0 & b \end{pmatrix}, \ \overleftrightarrow{R}_{A_2''}(z) = 0, \\
\overleftrightarrow{R}_{E_1'}(x) &= \begin{pmatrix} 0 & d & 0 \\ d & 0 & 0 \\ 0 & 0 & 0 \end{pmatrix}, \ \overleftrightarrow{R}_{E_1'}(y) = \begin{pmatrix} d & 0 & 0 \\ 0 & -d & 0 \\ 0 & 0 & 0 \end{pmatrix}, \\
\overleftrightarrow{R}^{(1)}_{\mathrm{E}''} &= \begin{pmatrix} 0 & 0 & 0 \\ 0 & 0 & c \\ 0 & c & 0 \end{pmatrix}, \quad \overleftrightarrow{R}^{(2)}_{E''} = \begin{pmatrix} 0 & 0 & -c \\ 0 & 0 & 0 \\ -c & 0 & 0 \end{pmatrix},
\end{aligned} \tag{9}$$

where a, b, c and d are constants. The Cartesian coordinates identify the polarization direction for polar modes (A_2 and E_1').

6. Raman scattering from phonon-polaritons

In order to conveniently model polariton scattering, Raman tensor elements shall be defined along normal polariton coordinates. Given the connection between polariton lattice deformation and macroscopic electric field, the resulting polariton tensor can be interchangeably expressed as a function of either their mechanical or electromagnetic components.

Polariton normal coordinates Polariton branches do not exhibit the same symmetries as the crystal lattice as they propagate obliquely in the crystal. In order to express the resulting first-order effect of the polariton on the susceptibility of the material, the polariton normal coordinates $\{To, Te, Le\}$ are projected onto the lattice coordinates $\{x, y, z\}$. With the polariton wavevector orientation in the lattice coordinates defined as $\boldsymbol{k} = k\,(\sin\theta\cos\phi, \sin\theta\sin\phi, \cos\theta)$, the polariton normal coordinates are defined such that $\widehat{To} \perp (\hat{z}, \boldsymbol{k})$, $\widehat{Te} \perp (\hat{To}, \boldsymbol{k})$ and $\widehat{Le} \parallel \boldsymbol{k}$.

Mechanical and electro-optical contributions of polaritons to the polarizability Raman scattering originates from the modulation of the susceptibility of a material by mechanical atomic displacements. However, oscillating macroscopic electric fields in a material can also modulate the susceptibility, which is fundamental premise of non-linear optics. Considering explicitly polar phonons or polaritons, the material susceptibility can be expressed as a Taylor series of the macroscopic electric field $\boldsymbol{E}$ associated to the lattice displacements. It is critical to note here that for polar vibrational modes, the lattice displacement and accompanying electric field possess the same symmetry and frequencies. Hence, the total first-order effect of the polariton on the material susceptibility can then be written as

$$\delta\overleftrightarrow{\chi}_N = \overleftrightarrow{R}_N Q_N + 2\overleftrightarrow{\chi}^{(2)}_N E_N, \tag{10}$$

with N indicating the polariton normal coordinates $\{To, Te, Le\}$, and Q_N and E_N the polariton displacements and electric fields. $\overleftrightarrow{\chi}^{(2)}$ is the second-order susceptibility tensor as usual defined in non-linear optics. Given their equivalent symmetry, Q_N and E_N can be linked by a frequency-dependent scalar parameter [25] :

$$E_{\perp,/\!/} = \frac{\omega^2_{\mathrm{TO};\perp,/\!/} - \omega^2}{\omega_{\mathrm{TO};\perp,/\!/}\sqrt{\epsilon_0\big(\epsilon_{s;\perp,/\!/} - \epsilon_{\infty;\perp,/\!/}\big)}} Q_{\perp,/\!/} = \frac{1}{K_{\perp,/\!/}} Q_{\perp,/\!/}, \tag{11}$$

defining $K_{\perp,/\!/} \equiv Q_{\perp,/\!/}/E_{\perp,/\!/}$. This relation establishes a direct proportionality between the electric and atomic displacement fields, and the variation of the susceptibility can then be

equivalently expressed as a function of the atomic displacements,

$$\delta\overleftrightarrow{\chi}_N \propto \left(\overleftrightarrow{R}_N + \frac{2}{K_N}\overleftrightarrow{\chi}_N^{(2)}\right) Q_N, \tag{12}$$

or as a function of the electric field,

$$\delta\overleftrightarrow{\chi}_N \propto \left(K_N\overleftrightarrow{R}_N + 2\overleftrightarrow{\chi}_N^{(2)}\right) E_N, \tag{13}$$

with $K_{To,Te} = K_\perp$ and $K_{Le} = K_\parallel$. Note here that K_N diverges for $\omega \to \omega_{TO}$, as the electric field vanishes at the transverse frequency.

These expressions demonstrate that the effective polariton Raman tensor can be expressed interchangeably as a function of the mechanical or electrical component, using the appropriate form of the tensor. This property enables the use of a numerical electromagnetic waveguide model to compute the Raman scattering efficiency. The effective polariton Raman tensor used here is defined as

$$\widetilde{R}_N \equiv K_N\overleftrightarrow{R}_N + 2\overleftrightarrow{\chi}_N^{(2)}. \tag{14}$$

The Faust-Henry coefficient The relative magnitudes of $\overleftrightarrow{R}_N$ and $\overleftrightarrow{\chi}_N^{(2)}$ for a given material can be expressed as a function of the Faust-Henry coefficients C_λ^{FH} [26]. As a function of the tensor elements, these coefficients are given by

$$\frac{R_{\lambda,ij}}{\chi_{\lambda,ij}^{(2)}} = C_{\lambda,ij}^{\mathrm{FH}} \frac{2\omega_{\mathrm{TO}\lambda}}{\sqrt{\epsilon_0(\epsilon_{s\lambda} - \epsilon_{\infty\lambda})}}, \tag{15}$$

where ϵ_s is the static relative permittivity.

7. Polariton scattering intensity

Taking into account the Hopfield coefficients h_λ^E [27] for each polariton coordinate, we can now calculate the Raman scattering efficiency using the polariton tensors presented above. These coefficients characterize the proportion of the energy in the electromagnetic and mechanical parts of the polariton. For uniaxial crystals, the expressions of these coefficients, expressed in the polariton coordinate basis, are given by [28],

$$H^E_{To}(\omega,\theta) = \frac{h^E_\perp}{\epsilon_{\infty\perp}}, \tag{16}$$

$$H^E_{Te}(\omega,\theta) = \frac{h^E_\perp \cos^2\theta}{\epsilon_{\infty\perp}} + \frac{h^E_{/\!/} \sin^2\theta}{\epsilon_{\infty /\!/}}, \tag{17}$$

$$H^E_{Le}(\omega,\theta) = \frac{h^E_\perp \sin^2\theta}{\epsilon_{\infty\perp}} + \frac{h^E_{/\!/} \cos^2\theta}{\epsilon_{\infty /\!/}}. \tag{18}$$

The total scattering intensity can then be expressed by including the explicit momentum conservation integral spanning from $-d/2$ to $d/2$ in the z dimension, which yields [29],

$$I_N(\omega) = \frac{C(n_\omega+1)}{d}\left|(\hat{e}_i\cdot\widetilde{R}_N\cdot\hat{e}_s)\int_{-d/2}^{d/2} e^{\iota\Delta\boldsymbol{q}_\perp z}\langle E_N(z)\rangle \mathrm{d}z\right|^2 \delta(\Delta\boldsymbol{q}_{/\!/}-k_{/\!/})H^E_N(\omega,\theta), \tag{19}$$

where $\Delta\boldsymbol{q} = \boldsymbol{q_i} - \boldsymbol{q_s}$, n_ω is the occupation factor of polaritons at frequency ω, $\langle E_N(z)\rangle = \sqrt{E_N(z)^\star E_N(z)}$ and C is a constant. The overlap integral between the polariton electric field distribution and the scattered momentum in the z axis, which results in a Dirac delta for the 'infinite' in-plane direction, gives rise to a relaxed z wave vector conservation condition in a thin film.

As a function of sample thickness, the Raman intensity is governed by the integral in Supplementary Equation 19 and the probed volume. At 532 nm, the integral is maximized for a thickness of about 150 nm. Away from this thickness, the integral slowly decreases. The probed volume increases linearly with thickness, but it saturates at the penetration depth of the excitation laser (5.6 µm).

8. Polariton Field distribution $E_N(z)$

In order to compute the Raman cross-section described by equation (19), the polariton electric field distribution inside the sample must be determined. The anisotropic multilayer media resulting from 2D material layering precludes the use of simple analytical approaches. The 4×4 transfer matrix formalism described in Ref. [30] provides a powerful and versatile numerical model of electromagnetic propagation through arbitrarily complex and anisotropic media.

Typical results are shown in Fig. 3, where the electric field spatial distribution $|E_x(z)|^2$ is presented as a function of frequency for four different tilt angles, corresponding to four

different in-plane wavevectors. For the purpose of this figure, the spectral broadening parameter is set to $\Gamma = 0.5$ cm^{-1} to help discriminate nearly-degenerate modes. Both surface polariton modes are clearly visible, as well as some lower and upper extraordinary polariton modes.

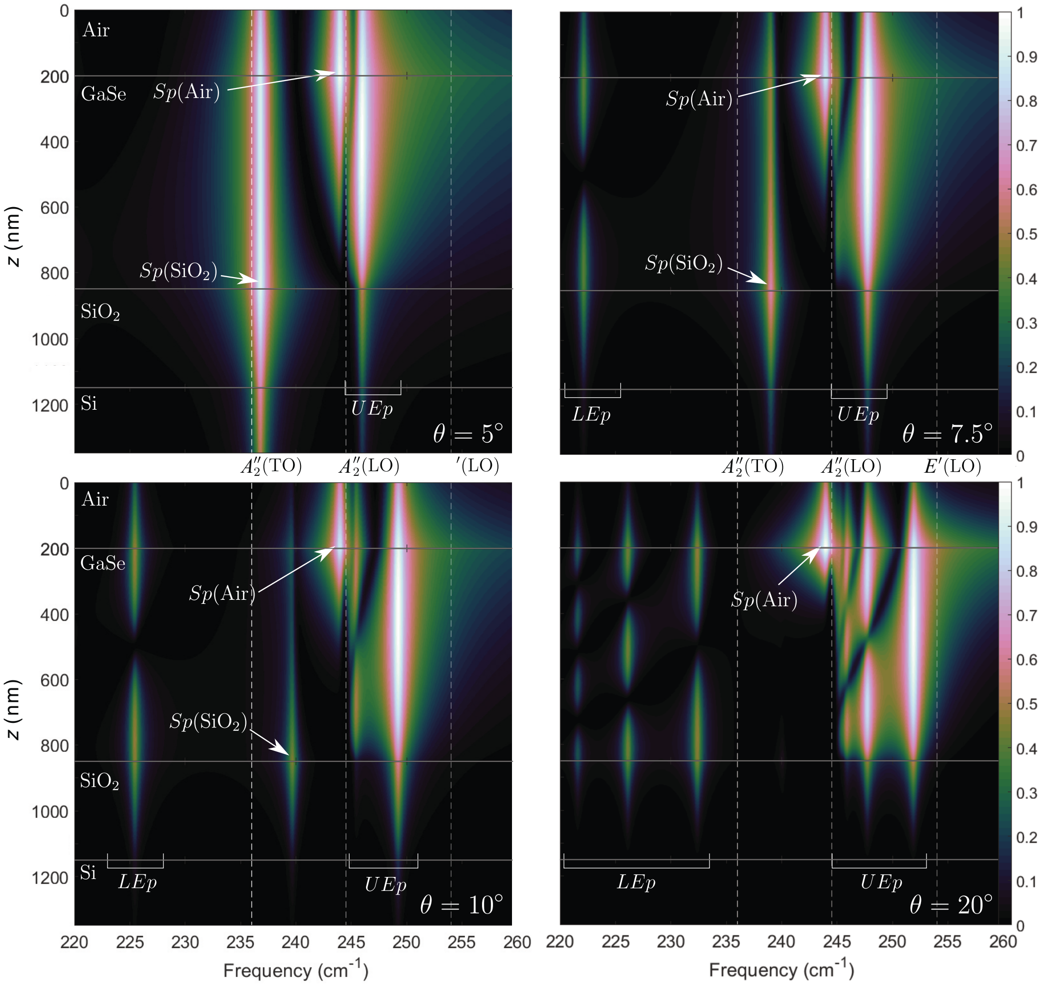

Supplementary Figure 3. Spatial field distribution in a 650 nm thick sample of GaSe on a Si/SiO_2 substrate for different scattering angles (θ = 5, 7.5, 10 and 20°). The field intensity $|E_x(z)|^2$ is presented using a logarithmic scale. The guided upper and lower extraordinary polariton branches are indicated by the white brackets and both surface polariton modes are indicated by Sp(Air) and Sp(SiO_2). Lattice normal modes are indicated by the vertical dashed lines.

9. Modelisation parameters

All parameters involved in Raman spectra calculations are taken from the literature, set by the sample structure, or determined by the measurement configuration. These values are presented in Supplementary Table 3. Except for an overall constant C used to adjust the absolute scattering intensity, no free parameters are required to compute the Raman spectra.

Supplementary Table 3. Parameters used in the computation of Raman spectra

Parameter	Description	Value
$C^{\mathrm{FH}}_{\lambda,ij}$	GaSe Faust-Henry coefficients	-0.3651 [29]
$\omega_{TO;\perp}$	GaSe $E'(TO)$ energy	$213.5\,\mathrm{cm}^{-1}$ [23]
$\omega_{TO;\perp}$	GaSe $E'(LO)$ energy	$254\,\mathrm{cm}^{-1}$ [23]
$\omega_{TO;/\!/}$	GaSe $A_2''(TO)$ energy	$236\,\mathrm{cm}^{-1}$ [23]
$\omega_{TO;/\!/}$	GaSe $A_2''(LO)$ energy	$244.5\,\mathrm{cm}^{-1}$ [23]
$\epsilon_{\infty;\perp}$	GaSe E' high-frequency permittivity	7.44 [23]
$\epsilon_{\infty;/\!/}$	GaSe A_2'' high-frequency permittivity	5.76 [23]
$\epsilon_{\mathrm{Si}}(\sigma)$	Silicon permittivity at σ	11.69 [31]
$\epsilon_{\mathrm{SiO_2}}(\sigma)$	SiO_2 permittivity at σ	Data from [32]
$\Gamma_{\perp,/\!/}$	Polariton broadening parameter	$1.8\,\mathrm{cm}^{-1}$
d_{GaSe}	GaSe slab thickness	As determined from AFM measurements
$d_{\mathrm{SiO_2}}$	Substrate SiO_2 thickness	300 nm
ϕ	Sample first Euler angle	0°
θ	Sample tilt (second Euler angle)	0-45°
ψ	Sample rotation (third Euler angle)	45°
σ	Probed polariton wavenumber	$180\text{-}265\,\mathrm{cm}^{-1}$
α	Laser polarization angle	45°

10. Calculated Raman backscattering intensity

The calculated polariton scattering intensity is shown in 4 as a function of $k_\parallel$ and polariton coordinates: (a) transverse ordinary (To), (b) transverse extraordinary (Te), and (c)

longitudinal extraordinary (Le).

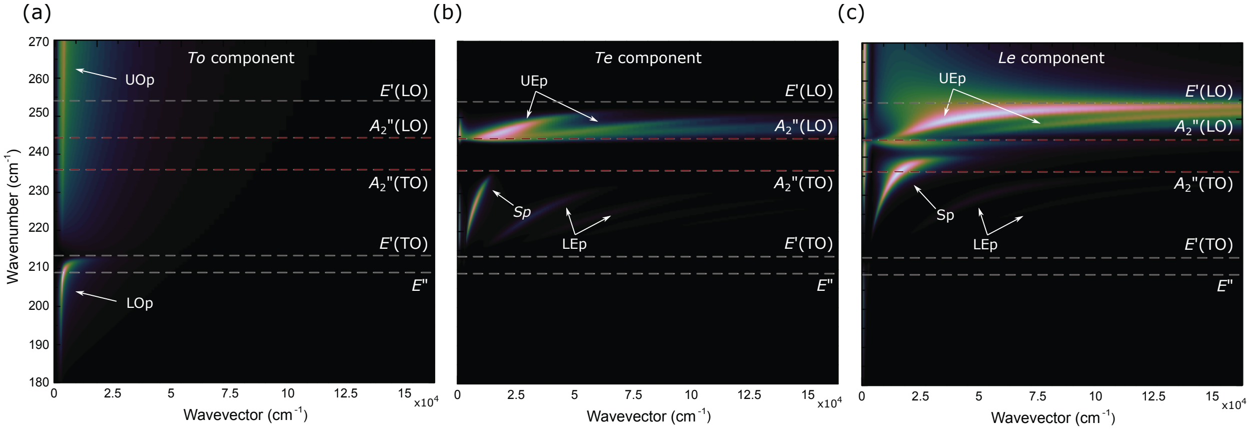

Supplementary Figure 4. Calculated polariton Raman scattering intensity dispersion for a 650 nm-GaSe sample on SiO_2/Si. (a-c) Calculated Raman spectra as a function of three normal coordinates (To, Te, Le).

11. Backscattering efficiency

The scattering anisotropy can be evaluated using the function $S = (I_F - I_B)/(I_F + I_B)$, where I_F and I_B are the forward and backward intensity integrated from 180 to 270 cm^{-1} and averaged over 15 logarithmically spaced scattering angles from 0 to 45°. Fig. 5 presents S as function sample thickness and reveals that for GaSe the anisotropy is negligible below 1 µm, $I_F \approx I_B$.

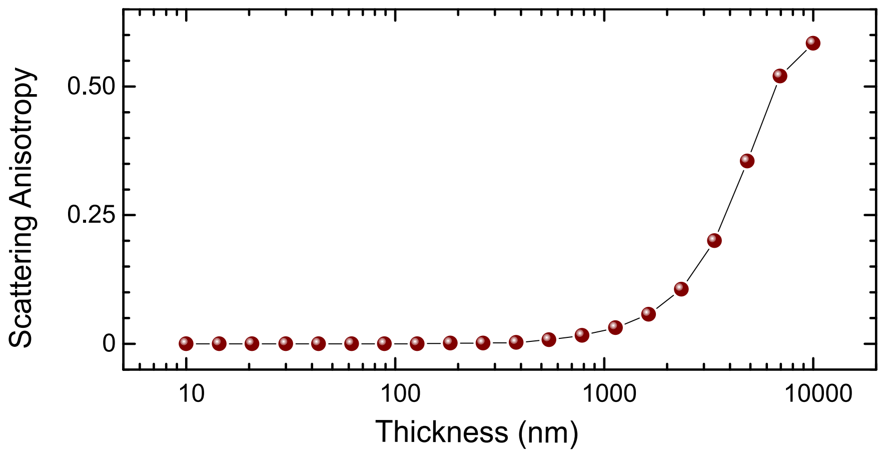

Supplementary Figure 5. Scattering anisotropy as a function of sample thickness. Below 1 µm, the anisotropy is negligible: forward and backward scattering are equality likely.

12. Forward scattering signal in backscattering configuration

For thick samples, the scattering anisotropy S is large (see Fig. 3(a)) and polaritons scattering is limited to very small wavevectors, hence preventing their observation in the typical backscattering configuration used for the study of 2D materials. We demonstrate below that polaritons can nonetheless be observed in backscattering configuration for thick samples in some special conditions.

Fig. 6 presents the Raman spectra of a freestanding 70 µm GaSe sample excited at 532 nm and 633 nm. In addition to the allowed lattice phonon at $\theta = 0$ (${A'_1}^1$, E'(TO), ${A'_1}^4$), the upper extraordinary polariton (UEp) is observed. Because of the weak confinement in a such a thick sample, this polariton appears slightly below the frequency of the forbidden E'(LO) phonon with an asymmetric lineshape extending towards low frequencies. It appears only when the sample is excited below the gap (633 nm), where the sample is transparent to both the incident and scattered light. In this condition, the penetration depth exceeds the sample thickness and the Raman spectra is composed of both a backscattered signal from lattice phonons ($k >> \omega/c$) and a forward-scattered signal from polaritons ($k \geq \omega/c$) involving a reflection at the back of the sample sending the light back toward the collection objective (see Fig. 1(c)). In both cases, the tangential polariton wave vector is the same, but there is

a significant difference in the magnitude of the probed perpendicular wave vector.

Backscattered and reflected forward scattered signals can be discriminated by moving the focal point of the 633 nm excitation along the optical axis. Figure 6(b) shows the integrated Raman intensity as a function of the focal point depth within the sample, whose surfaces are identified by the two vertical lines at 0 and 70 µm. The normalized intensity profiles of all three lattice phonons (${A'_1}^1$, ${A'_1}^4$, and E'(TO)) are almost identical and are maximum at about the center of the sample, as this position maximizes the probed volume (see middle inset). In contrast, directional forward scattering is expected to be minimum at the sample surface (see left insert) and maximum at the rear surface, where reflected rays are directly guided back to the collection optics (right inset). As can be seen from Fig. 6(b), the intensity profile of the upper extraordinary polariton (UEp) is very different from that observed from lattice phonons and indeed matches the profile expected from near-forward scattering involving a reflection at the back surface.

Hence, care must be applied in the assignment of phonons in 2D materials, as weakly confined polaritons may also contribute to Raman spectra even in a backscattering configuration. This aspect may help explain some of the confusion regarding the assignment of GaSe phonons in the literature.

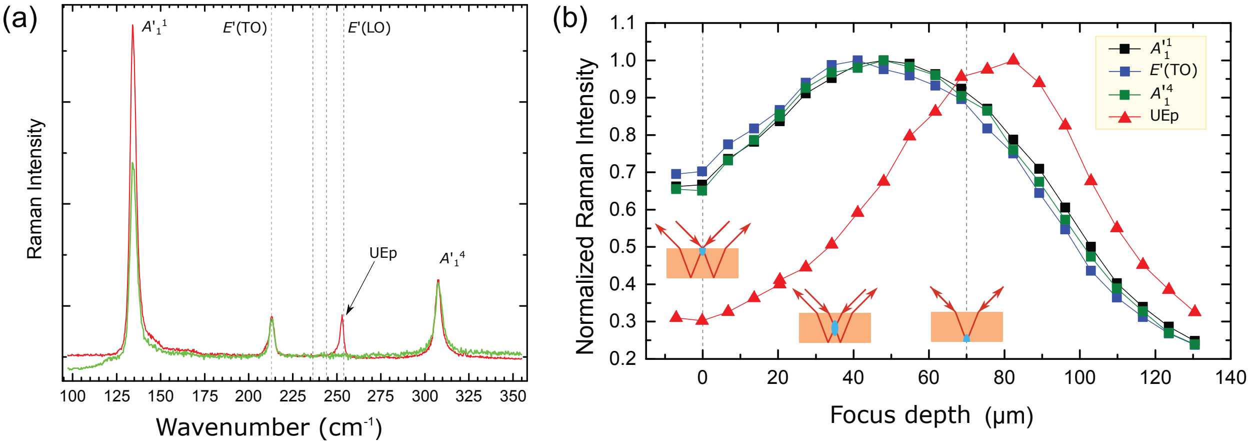

Supplementary Figure 6. (a) Backscattered Raman spectra from a freestanding 70 µm sample under 633 nm (red curve) and 532 nm (green curve) excitation at normal incidence. The lattice phonons (${A'_1}^1$, E'(TO) and ${A'_1}^4$) are readily identified along with the upper extraordinary polariton (UEp) mode at 252 cm^{-1}. The latter is observed only using sub-bandgap excitation (633 nm). (b) Normalized Raman backscattering intensity of the ${A'_1}^1$ (black squares), E'(TO) (blue squares), ${A'_1}^4$ (green squares) and UEp (red triangles), using a 633 nm excitation. Estimated locations of the sample interfaces are shown by the vertical lines. Insets: incident and scattered ray configurations at both surfaces and midway into the sample.

13. Raman spectra as a function of thickness

Interlayer interactions in thin 2D samples affect lattice phonon frequencies and, upon proper calibration, frequencies determined from Raman spectroscopy are commonly used for a convenient and rapid evaluation of sample thicknesses. Because of the relatively weak interlayer interactions, phonon frequency changes are typically resolved for samples composed of less than 20 monolayers. [33] In contrast, the thickness dependence of the surface and upper extraordinary polaritons shown in 7 allow extending the range where Raman scattering can be used for thickness measurements.

Polariton dispersion curves as a function of sample thickness are presented in 7 for different tilt angles θ. For simplicity, the effect of a finite angular acceptance of the instrumentation is not considered in the following discussion. For $\theta = 5°$, the lower surface polariton frequency sensitively depends on the thickness, spanning a range from about 220 cm^{-1} at 100 nm to about 240 cm^{-1} at 1000 nm. In contrast, the upper extraordinary polariton evolves

more slowly, starting at about 245 cm^{-1} and evolving to 250 cm^{-1}. At higher tilt angles, larger $k_{\|}$ are probed and the regions of rapid evolution of the polariton frequency are pushed to lower thicknesses.

This sensitive dependence of polariton frequencies could be exploited to determine sample thicknesses in a mesoscopic regime up to 1000 nm, thereby extending the range where Raman spectroscopy can be used to determine sample thickness by a few orders of magnitude.

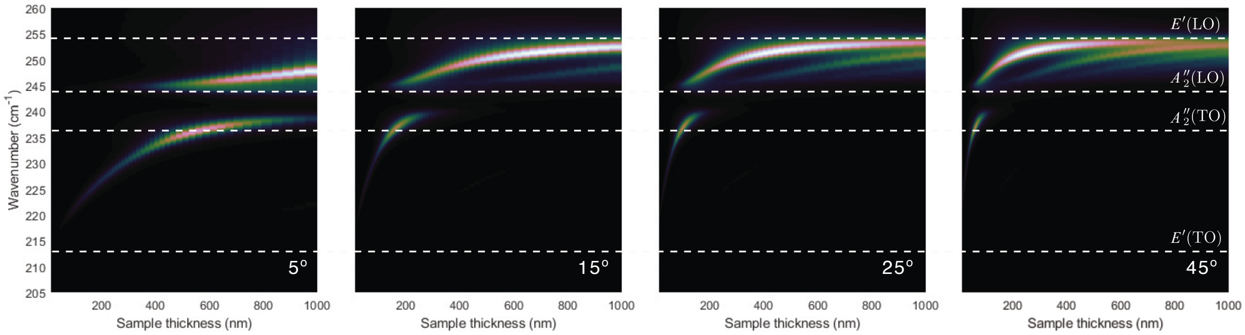

Supplementary Figure 7. Polariton Raman spectra as a function of sample thickness for various angles θ. The energies of the lattice phonons are indicated by the horizontal lines

14. Angular resolution

The finite angular aperture of the excitation and collection optics limits the capacity to resolve the dispersion relation of polaritons. Decreasing this aperture improves the resolution of the measurements. The simplest implementation consists in placing an iris in the optical path of the collimated Raman emission. For this demonstration, the LSp is selected for its relatively important dispersion at a sample tilt angle of $\theta = 10°$. Fig. 8 shows the the position and width of the LSp as function of the diameter of the iris. Reducing this diameter from 5 to 1 mm narrows the width from 10 to less than 7.5 cm^{-1}. Concomitantly, the position shifts to higher energy, indicating that the group velocity ($\frac{d\omega}{dk}$) decreases with the tilt angle (or $k_{\|}$).

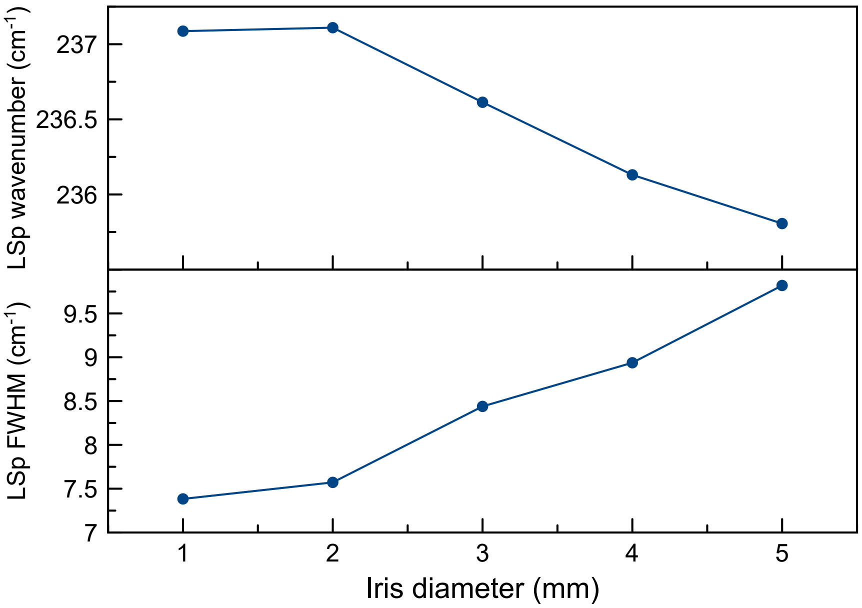

Supplementary Figure 8. Measure frequency and FWHM of the LSp in function of an iris diameter, placed before the focusing lens of the spectrometer. Measured from an estimated 270 nm thick GaSe sample.

SUPPLEMENTARY REFERENCES

---

* sebastien.francoeur@polymtl.ca

[1] R. M. Hoff, J. C. Irwin, and R. M. A. Lieth, Canadian Journal of Physics **53**, 1606 (1975).

[2] T. J. Wieting and J. L. Verble, Physical Review B **5**, 1473 (1972), ISSN 01631829.

[3] T. J. Wieting, Solid State Communications **12**, 931 (1973), ISSN 00381098.

[4] M. Hayek, O. Brafman, and R. M. A. Lieth, Physical Review B **8**, 2772 (1973).

[5] R. Le Toullec, N. Piccioli, and J. C. Chervin, Physical Review B **22**, 6162 (1980).

[6] V. K. Bashenov, I. Baumann, and D. I. Marvakov, Physica Status Solidi (b) **89**, K115 (1978).

[7] V. Y. Altshul, V. K. Bashenov, D. I. Marvakov, and A. G. Petukhov, physica status solidi (b) **98**, 715 (1980).

[8] T. J. Wieting and M. Schlüter, *Electrons and phonons in layered crystal structures*, vol. 3 (Springer, Dordrecht, Netherlands, 1979).

[9] S. Jandl and J. L. Brebner, Canadian Journal of Physics **52**, 2454 (1974).

[10] R. Longuinhos and J. Ribeiro-Soares, Physical Chemistry Chemical Physics **18**, 25401 (2016).

[11] N.M. Gasanly, A. Aydinli, H. Özkan, and C. Kocabas, Material Research Bulletin **37**, 169 (2002).

[12] K. Allakhverdiev, N. Fernelius, and F. Gashimzade, J. Appl. Phys. **93**, 5 (2003).

[13] V. Riede, H. Neumann, F. Lévy, and H. Sobotta, physica status solidi (b) **104**, 277 (1981).

[14] N. Kuroda, O. Ueno, and Y. Nishina, Physical Review B **35**, 3860 (1987).

[15] R. Le Toullec, J. C. Chervin, N. Piccioli, and A. Chevy, Applied Optics **20**, 2566 (1981).

[16] C. H. Henry and J. J. Hopfield, Physical Review Letters **15**, 964 (1965).

[17] R. Frech, The Journal of Chemical Physics **67**, 952 (1977).

[18] R. Claus, Physica Status Solidi b **50**, 11 (1972).

[19] R. Loudon, Advances in Physics pp. 423–482 (1964).

[20] L. Merten and G. Lamprecht, physica status solidi (b) **39**, 573 (1970).

[21] T. Kurosawa, Journal of the Physical Society of Japan **16**, 1298 (1961).

[22] C.-w. Chen, T.-t. Tang, S.-h. Lin, J. Y. Huang, C.-s. Chang, P.-k. Chung, S.-t. Yen, and C.-l. Pan, Journal of the Optical Society of America B **26**, A58 (2009).

[23] E. Palik, *Handbook of Optical Constants of Solids Vol. III* (Academic Press, 1997).

[24] R. Claus, L. Merten, and J. Brandmuller, *Light Scattering by Phonon-Polaritons*, vol. 75 of *Springer Tracts in Modern Physics* (Springer Berlin Heidelberg, Berlin, Heidelberg, 1975).

[25] P. Y. Yu and M. Cardona, *Fundamentals of Semiconductors : Physics and Materials Properties* (Springer-Verlag Berlin Heidelberg, 2010) , 4th ed., ISBN 978-3-642-00710-1.

[26] W. L. Faust and C. H. Henry, Physical Review Letters **17**, 1265 (1966).

[27] J. J. Hopfield, Physical Review **112**, 1555 (1958).

[28] G. Irmer, C. Röder, C. Himcinschi, and J. Kortus, Physical Review B - Condensed Matter and Materials Physics **88**, 104303 (2013).

[29] Y. Sasaki and S. Ushioda, Physical Review B **27**, 1122 (1983).

[30] N. C. Passler and A. Paarmann, Journal of the Optical Society of America B **34**, 2128 (2017).

[31] W. Karstens, D. C. Bobela, and D. Y. Smith, Journal of the Optical Society of America A **23**, 723 (2006).

[32] G. Cataldo, E. J. Wollack, A. D. Brown, and K. H. Miller, Optics Letters **41**, 1364 (2016).

[33] D. J. Late, B. Liu, H. S. S. R. Matte, C. N. R. Rao, and V. P. Dravid, Advanced Functional Materials **22**, 1894 (2012).
